# Molecular Insights in Endometrial Stromal Sarcomas: Exploring New Targets for Novel Therapeutic Approaches

**DOI:** 10.3390/biom15020265

**Published:** 2025-02-11

**Authors:** Alice Costa, Annalisa Astolfi, Livia Gozzellino, Margherita Nannini, Gianandrea Pasquinelli, Maria Abbondanza Pantaleo

**Affiliations:** 1 IRCCS Azienda Ospedaliero-Universitaria di Bologna, 40138 Bologna, Italy; alice.costa@aosp.bo.it (A.C.); annalisa.astolfi@unibo.it (A.A.); 2 Department of Medical and Surgical Sciences (DIMEC), Alma Mater Studiorum, University of Bologna, 40138 Bologna, Italy; livia.gozzellino2@unibo.it (L.G.); margherita.nannini@unibo.it (M.N.); maria.pantaleo@unibo.it (M.A.P.); 3 Division of Oncology, IRCCS Azienda Ospedaliero-Universitaria di Bologna, 40138 Bologna, Italy; 4 Division of Pathology, IRCCS Azienda Ospedaliero-Universitaria di Bologna, 40138 Bologna, Italy

**Keywords:** endometrial stromal sarcomas, molecular targets, therapeutic strategies

## Abstract

Uterine mesenchymal tumors (UMTs) are the second most common type of tumors within the uterus corpus after endometrial carcinomas. Among the UMTs, smooth muscle neoplasms are the most common subtype, followed by endometrial stromal sarcoma (ESS). ESSs are uncommon malignancies characterized by molecular heterogeneity and an aggressive behavior. Their management poses significant challenges, particularly for high-grade subtypes. Surgery is the primary intervention for localized disease, while the role of adjuvant therapies, including radiation and chemotherapy, must be better investigated. Hormonal therapy has shown efficacy in low-grade cases but limited success in high-grade tumors. Recent advancements in molecular profiling have revealed potential targets, offering promise for personalized treatments. However, novel therapeutic strategies are urgently needed to improve patient outcomes, particularly for advanced and recurrent disease. This review offers a perspective on the possible novel therapeutic approaches based on the most recent molecular analyses performed on endometrial stromal sarcomas.

## 1. Introduction

Uterine sarcomas (USs) are rare malignancies, accounting for approximately 1% of female genital tract cancers and 3–9% of all uterine neoplasms [1]. Within the uterus corpus, mesenchymal tumors (UMTs) are the second most prevalent subtype, making up 8% of uterine cancers. Among UMTs, smooth muscle tumors are the most frequently observed, with leiomyosarcomas (uLMs) accounting for 63% of USs, followed by endometrial stromal sarcomas, and other rarer entities that collectively constitute the remaining UMTs [2].

Uterine leiomyosarcoma is the most common type of US, characterized by a poor prognosis due to a high rate of recurrence and metastasis. The 5-year survival rate ranges from 25% to 76%, dropping to 10–15% for patients with metastatic disease at diagnosis. At the molecular level, it is characterized by recurrent alterations in *TP53*, *RB1*, *ATRX*, *PTEN*, and *MED12* [3]. Although targeted therapies addressing key biological pathways have shown promising results, the persistent challenge of chemotherapy resistance highlights the urgent need for more effective treatment options [4].

Other, less frequent subtypes include endometrial stromal sarcomas (ESSs), which account for 21% of the UMTs [1,2]. ESSs are particularly rare, representing less than 1% of all uterine neoplasms [5], with an annual incidence of about 0.30–0.36 per 100,000 women. These tumors predominantly affect peri- and postmenopausal women, with most diagnoses made at an early stage (stage I) [6,7].

Conventional treatments for ESSs primarily involve surgery, with total hysterectomy and bilateral salpingo-oophorectomy being the standard approach for low-grade ESS (LG-ESS), followed by adjuvant hormonal therapy to reduce recurrence risk. In contrast, adjuvant chemotherapy is not the standard care for high-grade ESS (HG-ESS) but may be considered in selected FIGO II-III cases with poor prognosis [8]. Adjuvant radiotherapy has not shown a significant survival benefit in early-stage high-grade sarcomas and is therefore not routinely recommended, though some retrospective data suggest it may help reduce local relapse in LG-ESS. Despite the established role of surgery as the cornerstone of ESS treatment, the limited efficacy of current adjuvant strategies underscores the need for alternative therapeutic approaches [8,9]. Histologically and molecularly heterogeneous, ESSs pose significant diagnostic and therapeutic challenges, hampering the efforts to standardize treatment and improve outcomes. Here, we aim to summarize the molecular insights of endometrial stromal sarcomas and explore potential new targets for novel therapeutic approaches.

## 2. Endometrial Stromal Sarcoma Classification

**Figure 1:**
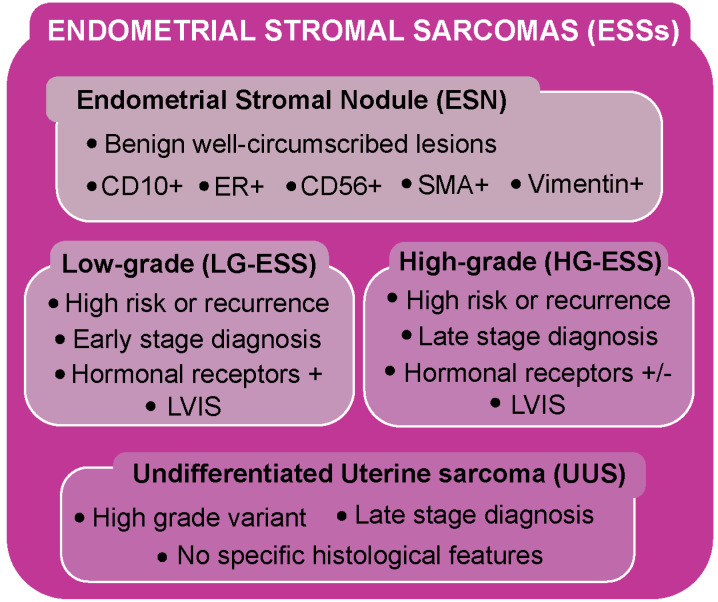
Overview of the main subtypes of endometrial stromal sarcoma. (LVIS: lympho-vascular invasion; ER: estrogen receptor; SMA: smooth muscle actin).

According to the 2020 World Health Organization (WHO) classification [10], ESSs are divided into four primary categories (Figure 1): benign endometrial stromal nodule (ESN), low-grade endometrial stromal sarcoma (LG-ESS), high-grade endometrial stromal sarcoma (HG-ESS), and undifferentiated uterine sarcoma (UUS) [11].

ESNs are benign well-circumscribed lesions, which closely resemble endometrial stromal cells during the proliferative phase of the menstrual cycle. These nodules typically show positive staining for CD10, estrogen receptor (ER), CD56, smooth muscle actin, and vimentin [5]. A key distinction between this type of lesion and LG-ESS is the presence of pushing margins in ESNs, along with the absence of lympho-vascular invasion (LVIS) [7]. However, a differential diagnosis between ESNs and LG-ESSs is not possible in curettage (a type of tissue scraping or removal from the uterus), making a precise diagnosis difficult. LG-ESS is the most common ESS subtype, typically affecting perimenopausal women. It tends to behave as a slow-growing tumor, with an overall 5-year survival rate of 80–90% for early-stage diagnoses. However, there is a high risk of multiple or late recurrences, with 15–25% of patients dying due to disease relapse [6,11,12]. LG-ESS typically exhibits infiltrative growth, LVIS, tongue-like margins, and high expression of estrogen receptor alpha (ERα) and progesterone receptor (PR), suggesting hormone dependence in many cases. Hormonal therapy (HT) with high-dose progestins or aromatase inhibitors is effective in treating LG-ESS, particularly in ER-positive metastatic cases [5,13,14].

In contrast, HG-ESS is a highly aggressive malignancy characterized by rapid progression, frequent recurrences, and a high tendency for metastasis, leading to a poor overall prognosis [5]. HG-ESSs were originally classified as undifferentiated stromal sarcomas in the 2003 WHO classification, and then redefined in 2014 following the discovery of the *YWHAE::NUTM2A/B* fusion, associated with the *t*(10;17)(q22;p13) chromosomal translocation [15,16,17]. Patients with HG-ESS typically have a median overall survival (OS) of 11–24 months, and the tumor is often diagnosed at advanced stages [6,11]. These neoplasms often exhibit hemorrhage and necrosis, with an infiltrative pattern of myometrial invasion and lympho-vascular space involvement. Unlike LG-ESSs, HG-ESSs generally lack hormone receptor expression (ER and PR), limiting the efficacy of hormonal therapies [5].

The last group of ESSs is represented by the undifferentiated uterine sarcoma (UUS), another high-grade variant, which predominantly affects postmenopausal women and represents less than 5% of all uterine sarcomas. It lacks specific histological features and is often diagnosed at an advanced stage, with a tendency to involve the peritoneum, lymph nodes, lungs, and bones. Like HG-ESS, UUS is highly aggressive and has a poor prognosis, with a median OS of less than 2 years in most cases [6,11,14].

Lymph node metastases in uterine sarcomas remain a complex and debated topic. Recent studies have highlighted that lymph node involvement is an independent prognostic factor, significantly reducing 5-year cause-specific survival in patients with positive nodes compared to those with negative nodes (30.4% versus 76.8% for ESSs) [18]. The likelihood of lymph node metastases varies across histological subtypes. For example, Nasioudis et al. reported lymph node positivity rates of 3.4% in leiomyosarcoma, 4.5% in LG-ESS, and comparable rates of approximately 7.9% in patients with HG-ESS and UUS [14]. However, these data predominantly refer to patients with early-stage disease, a limitation that should be carefully considered to avoid misleading interpretations. Current guidelines recommend lymphadenectomy only in cases with clinical or radiological suspicion of nodal involvement or confirmed HG-ESS. Therefore, systematic lymphadenectomy is not universally recommended and should be tailored to the individual patient based on clinical presentation and histological subtype [18].

## 3. Challenges in Treatment and Prognosis

Surgical management, usually involving hysterectomy, remains the cornerstone of treatment, regardless of the histotype; however, in cases of advanced or recurrent disease, surgery is often insufficient [19]. While hormonal therapy, such as high-dose progestins or aromatase inhibitors, has demonstrated effectiveness in treating LG-ESS, evidence supporting the use of chemotherapy or radiotherapy, especially in HG-ESS and UUS, is limited [5,6,11]. The most active chemotherapy regimens for advanced HG-ESS disease remain doxorubicin or a combination of gemcitabine and docetaxel, while radiotherapy is typically reserved for selected cases to alleviate symptoms [19].

Interestingly, a recent study developed a deep learning model called MMN-MIL to predict patient outcomes based on a small cohort of patients. The model achieved satisfactory prediction accuracy, making it a promising tool for predicting clinical outcomes in US patients and guiding early-treatment decisions [20].

Some recent research and clinical trials on therapeutic approaches in ESSs are shown in Table 1 and Table 2, respectively.

## 4. ESS Molecular Characterization: Implications for Diagnosis and Treatment

The molecular complexity of ESS plays a pivotal role in both diagnosis and therapeutic decision-making. In recent years, significant progress has been made in understanding the genetic landscape of uterine mesenchymal neoplasms, leading to the identification of novel entities and to a deeper characterization of already known tumor types [7].

The diagnosis of uterine sarcomas is particularly complex due to their rarity and heterogeneity. A comprehensive approach combining histological evaluation, immunohistochemistry, and molecular analysis is crucial, as outlined in the WHO Classification of Female Genital Tumors (5th edition) and ICCR (International Collaboration on Cancer Reporting) guidelines [10,31]. Immunohistochemistry aids in diagnosis and therapeutic decision-making by distinguishing between tumor subtypes, such as LG-ESS and UUS, based on markers like estrogen and progesterone receptors, p53, and CD10. These markers help guide treatment strategies, with Ki-67 providing prognostic insights into tumor aggressiveness [32].

Immunohistochemistry is useful in both diagnosis and therapeutic decision-making, while molecular analyses, such as RNA or DNA sequencing, are essential to identify fusion transcripts and mutations required for tumor classification and targeted treatment. Recent studies have revealed that specific gene rearrangements, like *JAZF1::SUZ12* for LG-ESS and *YWHAE::NUTM2A/B* for HG-ESS, are critical for accurate tumor classification. Advanced RNA and DNA sequencing techniques offer important insights into the tumor’s biological behavior and enhance diagnostic precision. When combined with immunohistochemical findings, these molecular analyses enable clinicians to tailor therapies more effectively, improving patient outcomes by identifying the most appropriate treatments based on the tumor’s unique genetic profile [7,20,32]. However, morphology remains the key for distinguishing subtypes like LG-ESS and HG-ESS, coupled to molecular analyses to refine diagnosis in challenging cases [33]. Moreover, molecular testing plays a decisive role in distinguishing HG-ESS from UUS, which are often diagnosed by exclusion after ruling out other uterine sarcomas [34].

**Figure 2:**
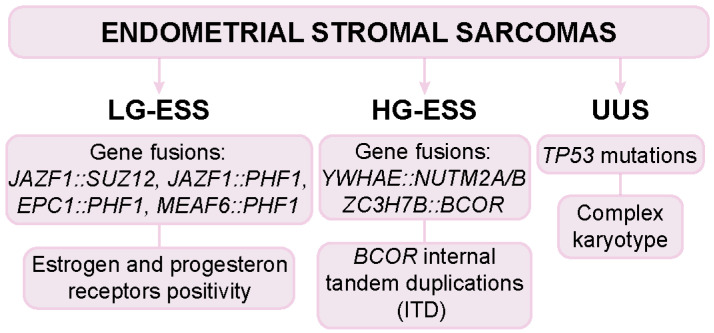
Key molecular markers and chimeric fusion genes useful for endometrial stromal sarcoma subtype classification. (LG-ESS: low-grade endometrial stromal sarcoma; HG-ESS: high-grade endometrial stromal sarcoma; UUS: undifferentiated uterine sarcoma).

Key molecular markers and chimeric fusion genes are critical for differentiating between ESS subtypes and for clinical decision-making (Figure 2) [35].

The majority of the newly recognized US genotypes are fusion-driven, while recurrent oncogene mutations are rare. In ESS, distinct molecular markers have emerged as key diagnostic tools. For example, *JAZF1::SUZ12*, *JAZF1::PHF1*, *EPC1::PHF1*, and *MEAF6::PHF1* fusions are commonly implicated in LG-ESS, while *YWHAE::NUTM2A/B* and *ZC3H7B::BCOR* fusions or *BCOR* internal tandem duplications (ITD) represent the main genetic abnormalities associated with HG-ESS [19,20,36]. These molecular features not only guide diagnosis but also represent potential therapeutic targets [35]. A recent study evaluated the diagnostic value of an IHC panel combining BCOR, Cyclin D1, and CD10 to distinguish ESS from other uterine lesions. The research highlighted that a strong BCOR nuclear expression in ≥95% of tumor cells, along with Cyclin D1 positivity and CD10 negativity, serves as a significant diagnostic feature for HG-ESS, with Cyclin D1 specifically positive in all HG-ESS cases and negative in all LG-ESS cases. The combination of a BCOR-positive, Cyclin D1-positive, and CD10-negative immunoprofile provides a highly specific, though less sensitive, diagnostic tool for identifying HG-ESS and differentiating it from other similar uterine lesions with overlapping histo-morphological features [37].

UUSs lack a specific immunophenotype, reflecting their heterogeneity, with CD10 being variably positive and hormone receptors usually weak or negative. They are also characterized by a complex karyotype with numerous chromosomal abnormalities, coupled with *TP53* mutations [38]. Among the subtypes of UUS, *SMARCA4*-deficient ones (SDUSs) represent a distinct entity characterized by the deletion of the *SMARCA4* gene, which leads to transcriptional and DNA damage repair dysregulation. Unlike other UUSs, SDUS lacks typical cancer markers such as cytokeratin, CD56, and EMA (epithelial membrane antigen), but expresses vimentin. Additionally, it is characterized by *SMARCA4* inactivation, which results in the loss of the transcription activator BRG1. SDUS shares morphological features with other aggressive tumors, including the small cell carcinoma of the ovary, hypercalcemic type (SCCOHT), but also presents distinguishing traits like its molecular profile and clinical presentation (e.g., cervical mass or vaginal bleeding). Advances in molecular diagnostics have highlighted the significance of *SMARCA4* loss in UUSs, refining their classification and underscoring the need for accurate diagnosis to guide therapeutic approaches [39].

In addition, UUS diagnosis is decreasing, as recurrent molecular alterations characterizing novel uterine sarcoma entities are identified. The standard treatment for UUS includes hysterectomy and bilateral salpingo-oophorectomy (BSO), while the role of lymphadenectomy remains controversial. Chemotherapy might be considered due to the risk of hematogenous spread and distant metastases [38].

Given the limited response of HG-ESS and UUS to conventional treatments, identifying and targeting specific molecular pathways could open new therapeutic avenues. Hormone receptor expression, particularly the presence of estrogen and progesterone receptors in LG-ESS, offers another promising area for therapeutic intervention, as these receptors are detected in approximately 70% and 90% of cases, respectively [6,40]. However, this strategy is less effective for HG-ESS and UUS due to inconsistent or absent receptor expression. For these aggressive tumor subtypes, adjuvant chemotherapy might be proposed, especially in the cases of UUS. For example, it can be considered for selected stage I patients with large tumors, high mitotic index, surgical morcellation, and good performance status after carefully weighing the risk–benefit ratio with the patient. These regimes usually include doxorubicin or epirubicin, with or without ifosfamide [6].

Thus, the treatment landscape for uterine stromal sarcomas, especially high-grade variants, remains challenging due to their aggressive nature and molecular heterogeneity. While surgery is often the primary treatment, the need for novel, targeted therapies is critical, with advances in molecular diagnostics paving the way for more personalized approaches to improve outcomes in these rare and aggressive neoplasms.

## 5. Therapeutic Approaches in Endometrial Stromal Sarcomas

### 5.1. Hormonal Therapies

Hormonal therapy represents a key approach in managing LG-ESSs, as these neoplasms frequently express steroid hormone receptors, especially estrogen and progesterone receptors. Progestins, such as medroxyprogesterone acetate (MPA), have proven effective for palliative management in case of advanced disease, providing clinical benefit by slowing tumor progression. Aromatase inhibitors (AIs), which reduce estrogen production, are also widely used as first- and second-line treatments for LG-ESSs, offering a favorable tolerability profile and prolonged disease control in hormone receptor-positive tumors. Recent studies have further confirmed the efficacy of hormonal treatments in managing LG-ESSs. These therapies can also be employed as adjuvant treatments after surgery, helping to manage residual disease or prevent recurrence [24,41]. Given the typically indolent nature of LG-ESS and its responsiveness to hormonal manipulation, MPA and AIs play a key role in patient management. Due to the rarity of these neoplasms, only a limited number of case reports and small retrospective studies have been published, and no global consensus exists on postoperative treatment. However, available retrospective studies on adjuvant progestin therapy suggest a lower recurrence rate across all stages in patients receiving these drugs [40]. Progestin treatment for recurrent or metastatic LG-ESS has been reported in several case reports and retrospective studies, with a total clinically effective rate of 86.9%, and recent studies have shown a significant reduction in mortality to below 10%, largely attributed to the use of hormonal treatment [12]. Thus, hormonal therapies remain an important strategy for low-grade tumors, offering long-term disease control with few side effects.

Although hormone receptor expression, particularly estrogen and progesterone receptors, offers therapeutic potential in LG-ESS, this strategy is poorly effective in HG-ESS and UUS due to the lack of steroid hormone receptors. However, recent evidence suggests that *BCOR*-related HG-ESS cells might variably express estrogen receptors, indicating that a combination of CDK4/6 inhibitors and aromatase inhibitors should be considered for ER-positive, *BCOR*-related metastatic HG-ESS patients [6]. Nevertheless, *ESR1* downregulation in HG-ESS, even in ER/PR-positive tumors, poses challenges for endocrine therapies, limiting their effectiveness [42].

While hormonal therapy offers benefits in treating uterine sarcomas, there are several limitations. Although it has an acceptable toxicity profile, side effects such as gastrointestinal reactions, severe depression, weight gain, and thromboembolism can occur, especially in the case of long-term high-dose progestin therapy. Additionally, the rarity of uterine sarcomas limits the available evidence on the effectiveness of hormonal treatments, and the optimal regimen remains uncertain. AIs, also, can lead to side effects like somnolence, rash, nausea, and fever due to interference with adrenal hormone production. Furthermore, the duration of hormonal therapy and the efficacy of combining different hormonal drugs are not well established [12,40].

### 5.2. Targeted Therapies

**Figure 3:**
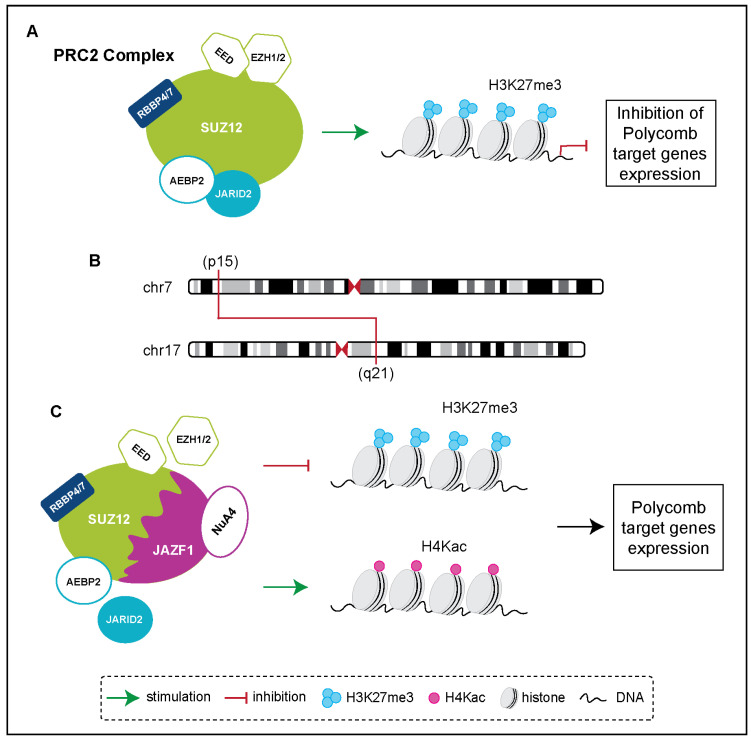
(**A**) The PRC2 complex trimethylates histone 3 on lysine 27 (H3K27me3), leading to the inhibition of Polycomb target genes expression. (**B**) Schematic representation of the [t(7;17)(p15;q21)] translocation, which generates a JAZF1::SUZ12 fusion protein, (**C**). This fusion disrupts SUZ12 interactions with EZH1/2 and JARID2, resulting in decreased H3K27me3 and increased H4 acetylation (H4Kac), ultimately leading to the activation of Polycomb target genes.

Recent research has shed light on crucial genetic mutations in uterine sarcomas that are pivotal for developing targeted therapies. As previously mentioned, *JAZF1* rearrangement is the most common molecular alteration in LG-ESS. The *JAZF1* gene can rearrange with various partners, more frequently with *SUZ12*. About 80% of LG-ESSs feature a [*t*(7;17)(p15;q21)] translocation [43]. This chromosomal rearrangement produces a JAZF1::SUZ12 fusion protein, resulting in disruption of the SUZ12 subunit of PRC2 (Polycomb Repressor Complex 2), which leads to PRC2 function dysregulation. Consequently, this results in reduced H3K27me3 (trimethylation of lysine 27 on histone H3) and increased H4Kac (lysine acetylation on histone 4) at target genes (Figure 3). The *JAZF1::SUZ12* fusion induces the ectopic activation of Polycomb target genes, which affect cell differentiation and adhesion, while inhibiting immune-related gene expression and promoting retention of cells in the endometrium [44]. Notably, Wnt activation, likely driven by overexpression of Wnt ligands, is a significant consequence of the *JAZF1::SUZ12* fusion. Interestingly, several of the less commonly observed fusions in LG-ESS, such as *JAZF1::PHF1* and *EPC1::PHF1*, also involve PRC2 complex subunits. This implies the occurrence of a common downstream epigenetic pathway among the gene fusions identified in LG-ESSs. Overall, these findings provide a compelling biological basis to explore Wnt pathway inhibitors, suggesting they could play a significant role in the treatment of LG-ESS [43,45,46]. Thus, the described gene fusions not only contribute to LG-ESS diagnosis but also provide potential targets for future therapeutic interventions.

Mutations in *ESR1*, linked to resistance to hormone therapies like aromatase inhibitors, represent another important target. Recent studies have documented the emergence of *ESR1* p.Y537S hotspot mutations in LG-ESS following hormonal therapy. *ESR1* encodes for the estrogen receptor, and these types of activating mutations enable coactivator binding even in the absence of estrogen, contributing to acquired endocrine resistance [47]. Notably, similar mutations have been identified in other cancers like breast cancer, particularly after hormonal therapy failure [48]. Additionally, other mechanisms of resistance, such as *ESR1* amplification, alterations in receptor tyrosine kinases, and pathways like *PI3K* and *MAPK*, have also been implicated in resistance to hormonal therapies. In such cases, fulvestrant, as many other selective estrogen receptor degraders (SERDs), has shown effectiveness, suggesting a promising targeted approach for treating recurrent tumors [25,49,50].

**Figure 4:**
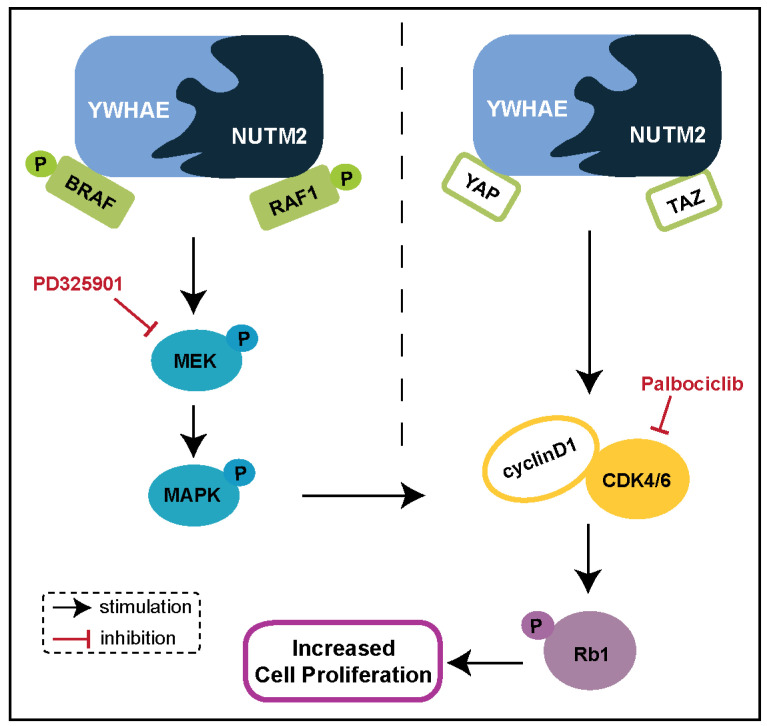
Schematic representation of the mechanisms by which YWHAE::NUTM2 fusion protein regulates cyclin D1 expression and cell proliferation in HG-ESS by dysregulating RAF/MEK/MAPK and Hippo/YAP-TAZ signaling pathways.

In contrast, HG-ESSs, exhibiting more aggressive behavior, are often associated with *YWHAE::NUMT2A/B* gene fusion. This fusion leads to the production of the 14-3-3 oncoprotein, which is implicated in tumorigenesis and represents a potential therapeutic target [43,51]. This complex interacts with crucial signaling pathways, including RAF/MEK/MAPK and Hippo/YAP-TAZ, both of which are essential for cell proliferation (Figure 4). Knockdown of *YWHAE::NUTM2* in HG-ESS models resulted in the inhibition of RAF/MEK/MAPK phosphorylation, downregulation of cyclin D1, and a subsequent reduction in cell proliferation. Moreover, silencing cyclin D1 in HG-ESS led to dephosphorylation of RB1 and inhibited tumor growth, highlighting the role of cyclin D1 in cell cycle regulation. In line with these findings, MEK inhibitors (e.g., PD325901) and CDK4/6 inhibitors (e.g., palbociclib) demonstrated antiproliferative effects in HG-ESS, with combined therapies showing synergistic activity [52,53]. This suggests that targeting these pathways could offer promising therapeutic efficacy.

Additionally, endometrial stromal sarcomas with *BCOR* rearrangements usually exhibit *MDM2* amplification and activation of the cyclin D1-CDK4 pathway. These rearrangements might indirectly promote the amplification of *MDM2* and *CDK4* by disrupting cellular signaling, leading to increased cell proliferation. This includes cyclin D1 overexpression or *CDKN2A* deletion, which further enhances CDK4 activation and facilitates the G1-to-S phase transition of the cell cycle [17,52]. These genetic alterations closely resemble the genomic profile of other sarcomas that respond to CDK4/6 inhibition, suggesting a potential therapeutic strategy involving CDK4/6 inhibitors, either as a single agent or in combination with MDM2 inhibitors, for *BCOR*-fusion-positive sarcomas [54].

Moreover, the Hippo/YAP-TAZ signaling pathway, which is frequently dysregulated in sarcomas, plays a critical role in HG-ESS (Figure 4) [53]. The activation of YAP and the increased expression of extracellular matrix (ECM)-related genes are linked to poor prognosis, underlining the importance of the tumor microenvironment in disease progression [55]. These findings open new avenues for treatments targeting YAP and other ECM regulators, potentially improving clinical outcomes for patients with HG-ESS. Moreover, the inhibition of the MAPK and PI3K-AKT signaling pathways has shown promising results in HG-ESS with *YWHAE::NUTM2A/B* fusion, suggesting that targeting these pathways could be an effective therapeutic strategy [16].

Gene expression profiling analyses on HG-ESS harboring *YWHAE* and *BCOR* fusions or ITD have demonstrated increased levels of *NTRK3*, *FGFR3*, and *RET*, implying a potential role of tyrosine kinase inhibitors, such as pazopanib and imatinib, in the treatment of high-grade ESS subtypes. Moreover, increased expression of *GLI1* and *PTCH1* has been observed, suggesting a potential rationale for exploring targeted inhibitors of the Sonic hedgehog pathway [45].

Other significant molecular features of HG-ESSs include c-KIT mutations, *PDGFR* alterations, and Wnt signaling pathway dysregulation. These factors also represent potential therapeutic targets, as they are involved in key processes such as cell proliferation, survival, and metastasis. In case reports and series, therapies targeting these molecular pathways have shown clinical responses. An example is the positive response of a patient with a *YWHAE::NUTM2* translocation and high c-KIT expression to the VEGFR, PDGFR, and c-KIT inhibitor pazopanib. Additionally, imatinib mesylate, which targets c-KIT, has shown efficacy in treating both high-grade and low-grade c-KIT-positive ESSs, further supporting the therapeutic potential of targeting c-KIT in these neoplasms [16].

In addition, a novel subtype of highly aggressive HG-ESS has recently been described presenting *ERBB2/ERBB3* mutations and positive expression of *S100* and *SOX10*. The origin and proper classification of this unclassified sarcoma still need to be verified, as well as the potential advantage of targeting ERBB2/ERBB3 tyrosine kinase mutations. However, the inclusion of *SOX10* as a screening tool in high-grade unclassified uterine sarcomas could enhance recognition of this entity, especially in putative cases of undifferentiated uterine sarcomas, and could enhance the precision of the diagnosis and treatment strategies [56].

### 5.3. Immunotherapy

The potential of immunotherapy in the treatment of uterine stromal sarcomas, particularly endometrial stromal sarcomas (ESSs), is a promising area of research. Tumors that often carry high mutational burdens or express specific immune markers, like PD-L1, might be candidates for immune checkpoint inhibition. A key aspect for enhancing immunotherapy is the use of genomic profiling, which is increasingly recognized as an important tool to identify patients more likely to benefit from such therapies. Recent studies investigated the potential of both targeted therapies and immunotherapy for ESS patients. Gene expression analyses highlighted a significant activation of immune-related pathways in HG-ESS, suggesting that these patients could benefit from immunotherapy [57]. Additionally, analysis of the immune microenvironment revealed varying levels of immune cell infiltration, with some patients showing potential responsiveness to immune checkpoint inhibitors (ICIs) like PD-L1-targeted therapies. However, the degree of immune infiltration and response depends on specific tumor characteristics, such as the presence of particular fusion genes underscoring the complexity of the tumor immune landscape [57]. In uterine carcinosarcomas, PD-L1 expression has been more extensively investigated, and ICIs are already considered potential treatments, even if it is known that uterine carcinosarcomas actually belong to the high-grade endometrial carcinomas category [58]. Among uterine sarcomas, the prevalence of PD-L1 expression varies significantly across subtypes. Recent research highlights the expression of immune checkpoint proteins, such as PD-L1, PD-1, CTLA-4, and IDO, in uterine mesenchymal tumors, including endometrial stromal sarcomas. PD-L1 and CTLA-4 are expressed in approximately 25% and 13% of ESSs, respectively. Interestingly, immune checkpoint protein expression was observed to be mutually exclusive between tumor cells and infiltrating lymphoid cells, suggesting distinct immunological dynamics [59]. Additionally, recent studies link *SMARCA4* loss to an enhanced immune response in specific uterine sarcoma subtypes. This finding highlights the potential vulnerability of these tumors to immune checkpoint blockade therapy, as *SMARCA4*-deficient sarcomas often display increased infiltration of immune cells such as CTLs and helper T cells at tumor margins [27,60].

The evolving landscape of immunotherapy for ESSs leverages their unique molecular characteristics, including recurrent chromosomal translocations that produce tumor-specific fusion proteins acting as neoantigens. This provides opportunities for immunotherapeutic interventions such as vaccines aimed at eliciting immune responses and reducing recurrence risk, as well as adoptive T cell therapies targeting these fusion proteins to reduce the tumor burden and establish durable immune memory. While ongoing clinical trials are critical to evaluate the safety and efficacy of these strategies, understanding the ESS immune microenvironment could further enhance therapeutic applications [61].

All together, these findings suggest that integrating factors like PD-L1 expression, immune infiltration, and specific genomic biomarkers could help tailor treatment approaches for ESS patients, particularly those with high-grade tumors. Currently, therapeutic approaches involving checkpoint inhibitors, cytokine therapy, and immune cell therapy are not being extensively investigated for HG-ESS. However, given the positive outcomes observed in other stromal cell tumors and uterine cancers, the potential of immunotherapy remains highly promising [17].

### 5.4. Chemotherapy and Novel Target Therapies

In the case of Stage I LG-ESSs, adjuvant therapy is not routinely adopted. The standard approach involves observation alone following surgical resection, given the typically favorable prognosis at this early stage. For recurrent LG-ESSs, hormonal therapy is recommended, as treatments like progestins and aromatase inhibitors have shown long-term benefits in limiting advanced and recurrent disease. Even after progression following first-line endocrine therapy, second-line hormonal treatments can still provide extended disease control [62]. However, if the neoplasm continues to progress despite multiple hormonal therapies, chemotherapy, including doxorubicin-based therapies or gemcitabine with docetaxel, is the subsequent treatment option [6]. The same chemotherapy regimens are commonly used for metastatic or recurrent HG-ESS and UUS, despite the widespread chemoresistance of these tumors. First-line therapy typically involves anthracyclines, either as monotherapy or in combination with ifosfamide or dacarbazine. Another effective regimen includes the combination of gemcitabine and docetaxel, while other potentially active agents include trabectedin, dacarbazine, and eribulin [40]. Persistent or recurrent HG-ESSs exhibit a poor response to chemotherapy, with effectiveness ranging from only 5% to 27% in cases of recurrence following first-line treatment [38]. To overcome chemoresistance, new therapeutic approaches have been investigated, comprising chemotherapy combined with targeted agents such as pazopanib, a multi-targeted receptor tyrosine kinase inhibitor. Pazopanib has demonstrated modest activity in clinical trials, offering a potential strategy to improve outcomes in these resistant cases [63].

Additionally, apatinib, an antiangiogenic agent targeting VEGF receptors, has recently demonstrated promising results in stabilizing disease and improving survival when combined with chemotherapy [26,64]. Trabectedin, another targeted agent, has been used effectively in combination with radiotherapy, providing long-term disease control in some HG-ESS patients [29].

In uLMSs, the presence of *BRCA2* somatic mutations has been associated with homologous recombination deficiency (HRD) and genomic instability. This genetic alteration points to the potential utility of PARP inhibitors as targeted therapies. These findings highlight the importance of further investigating homologous recombination repair (HHR) deficiencies across uterine sarcoma subtypes [65,66]. For example, combined therapies targeting DNA repair mechanisms, such as temozolomide coupled with olaparib, have shown efficacy against advanced uLMSs, particularly in patients harboring *BRCA* and *TP53* mutations [66,67]. This therapeutic strategy might also be relevant for other uterine sarcomas with similar genetic alterations. For instance, UUS frequently exhibit *TP53* mutations, further supporting the potential benefit of DNA repair-targeting therapies also for these tumors [68]. Combined therapies like temozolomide and olaparib offer a new treatment option for patients with aggressive uterine sarcomas, which are often resistant to conventional treatments. Thus, while chemotherapy is crucial for HG-ESSs, the integration of targeted agents provides a promising direction to enhance treatment efficacy and manage these aggressive tumors.

## 6. Conclusions

Endometrial stromal sarcomas are challenging malignancies due to their molecular heterogeneity, aggressive nature, and limited therapeutic options. Despite the progress in understanding their genetic and molecular profiles, high-grade variants like HG-ESS and UUS pose significant diagnostic and therapeutic difficulties. Hormonal therapies, such as progestins and aromatase inhibitors, are effective for LG-ESSs, but show limited efficacy in high-grade tumors. Fusion genes and mutations in key pathways like CDK4/6, PI3K/AKT, and MAPK offer potential therapeutic targets for personalized treatments. However, the need for novel pharmacological strategies remains urgent, as conventional therapies, including chemotherapy and radiation, often show limited efficacy. Immunotherapy, particularly for tumors with a high mutational burden or PD-L1 expression, shows promising results, but further research is required to select potential responders.

Given the diagnostic complexity and rarity of this rare subset of mesenchymal uterine tumors, a routine integration of next-generation sequencing (NGS) analysis is highly recommended. The use of targeted genetic panels to identify relevant mutations and gene fusions in these diagnostically challenging cases could significantly enhance the accuracy of conventional diagnostics. Systematic molecular profiling in all ESS diagnoses could not only support or refine the diagnostic classification but also guide the selection of targeted molecular therapies.

Overall, while surgery remains the cornerstone of treatment for uterine sarcomas, integrating molecular profiling and targeted therapies could improve outcomes for patients with these rare and aggressive malignancies.

## Figures and Tables

**Table 1:** Recent research on therapeutic approaches in ESSs.

Year	Study Type	ESS Subtype	Therapy	Ref.
2019	Retrospective study	LG-ESS	Adjuvant hormonal therapy	[21]
2021	Case report	LG-ESS	Aromatase inhibitor	[22]
2022	Case report	Recurrent HG-ESS	PTX and CBDCA	[23]
2022	Retrospective study	Stage II-IV LG-ESS	Hormone therapy	[24]
2022	Retrospective study	LG-ESS	Surgical approaches	[13]
2022	Research report	LG-ESS	Aromatase inhibitors	[25]
2023	Perspective phase II study	HG-ESS	Apatinib	[26]
2023	Review article	LG-ESS, HG-ESS, and UUS	Surgical approaches	[18]
2024	Case report	SDUS	Immunotherapy	[27]
2024	Case report	HG-ESS	PARPi and anti-PD1	[28]
2024	Case report	HG-ESS	Trabectedin and radiotherapy	[29]
2024	Case report	LG-ESS	Aromatase inhibitor, MPA, doxorubicine, isofosfamide, pazopanib	[30]

Abbreviations: ESS, endometrial stromal sarcoma; LG, low-grade, HG, high-grade; SDUS, *SMARCA4*-deficient undifferentiated sarcoma; PTX, paclitaxel; CBDCA, carboplatin; MPA, medroxyprogesterone acetate.

**Table 2:** Ongoing clinical trials in ESS.

Phase	Title	ESS Subtype	Status	NTC Identifier
Observational	Prognosis of Low-grade Endometrial Stromal Sarcoma	LG-ESS	Recruiting	NCT05310318
I	A Study of Different Dosing Schedules of Selinexor in Sarcoma Patients	ESS	Active, not recruiting	NCT04811196
II	Phase II Trial of Single-Agent Nivolumab in Patients With Microsatellite Unstable/Mismatch Repair Deficient/Hypermutated Uterine Cancer	UUS, HG-ESS	Completed	NCT03241745
II	An Open-Label, Single-Arm, Prospective, Multi-Center, Tandem Two- Stage Designed Phase II Study to Evaluate the Efficacy of Fulvestrant in Women With Recurrent/Metastatic Estrogen Receptor-Positive Gynecologic Malignancies	ESS	Completed	NCT03926936
II	Evaluation of Clinical Impact of Interruption VS Maintenance of AI in Patients With Locally Advanced/Metastatic Low Grade Endometrial Stromal Sarcoma (LGESS) (BFR-ESS)	LG-ESS	Recruiting	NCT03624244
II	Tailoring Therapy in Post-surgical Patients With Low-risk Endometrial Cancer	Uterine Corpus ESS	Recruiting	NCT06388018
II	FUlvestrant in Gynecological Cancers That Are Potentially Hormone Sensitive: the FUCHSia Study (FUCHSia)	ESS	Completed	NCT03926936
II	A Study of Nivolumab in Selected Uterine Cancer Patients	HG-ESS	Completed	NCT03241745

Abbreviations: ESS, endometrial stromal sarcoma; LG, low-grade, HG, high-grade; UUS, undifferentiated uterine sarcoma.

## Data Availability

Not applicable.
